# Novel flowcytometry-based approach of malignant cell detection in body fluids using an automated hematology analyzer

**DOI:** 10.1371/journal.pone.0190886

**Published:** 2018-02-09

**Authors:** Tomohiko Ai, Yoko Tabe, Hiroyuki Takemura, Konobu Kimura, Toshihiro Takahashi, Haeun Yang, Koji Tsuchiya, Aya Konishi, Kinya Uchihashi, Takashi Horii, Akimichi Ohsaka

**Affiliations:** 1 Department of Next Generation Hematology Laboratory Medicine, Juntendo University Graduate School of Medicine, Tokyo, Japan; 2 Department of Clinical Laboratory Medicine, Juntendo University Graduate School of Medicine, Tokyo, Japan; 3 Department of Clinical Laboratory, Juntendo University Hospital, Tokyo, Japan; 4 Sysmex, Hematology-Product Engineering, Product Development, Kobe, Japan; 5 Department of Transfusion Medicine and Stem Cell Regulation, Juntendo University Graduate School of Medicine, Tokyo, Japan; Universidade Nova de Lisboa Instituto de Higiene e Medicina Tropical, PORTUGAL

## Abstract

Morphological microscopic examinations of nucleated cells in body fluid (BF) samples are performed to screen malignancy. However, the morphological differentiation is time-consuming and labor-intensive. This study aimed to develop a new flowcytometry-based gating analysis mode “XN-BF gating algorithm” to detect malignant cells using an automated hematology analyzer, Sysmex XN-1000. XN-BF mode was equipped with WDF white blood cell (WBC) differential channel. We added two algorithms to the WDF channel: Rule 1 detects larger and clumped cell signals compared to the leukocytes, targeting the clustered malignant cells; Rule 2 detects middle sized mononuclear cells containing less granules than neutrophils with similar fluorescence signal to monocytes, targeting hematological malignant cells and solid tumor cells. BF samples that meet, at least, one rule were detected as malignant. To evaluate this novel gating algorithm, 92 various BF samples were collected. Manual microscopic differentiation with the May-Grunwald Giemsa stain and WBC count with hemocytometer were also performed. The performance of these three methods were evaluated by comparing with the cytological diagnosis. The XN-BF gating algorithm achieved sensitivity of 63.0% and specificity of 87.8% with 68.0% for positive predictive value and 85.1% for negative predictive value in detecting malignant-cell positive samples. Manual microscopic WBC differentiation and WBC count demonstrated 70.4% and 66.7% of sensitivities, and 96.9% and 92.3% of specificities, respectively. The XN-BF gating algorithm can be a feasible tool in hematology laboratories for prompt screening of malignant cells in various BF samples.

## Introduction

Differentiation of nucleated cells including malignant cells in various body fluid (BF) samples is an essential technique to determine the clinical treatment strategies. A positive effusion for malignant cells is an important indicator in the diagnosis of malignant lesions and staging [1]. Therefore, the examination of BF for the presence of malignant cells has been accepted as a routine laboratory procedure, not only for the detection of incidental malignancy, but also for the detection of metastasis of an unknown primary origin [1, 2]. Especially, cytological examinations with papanicolaou and immunohistochemical stainings performed in pathology laboratories are of paramount importance in the diagnosis of malignancy in BF samples [2–4]. However, the routine cytology results are not available in the same day when the samples are sent to the lab, which prevents physicians from making a quick diagnosis. Hence, it is expected that the screening of malignant cells by the hematological examinations enables a rapid report to physicians and might be useful as adjunct rapid diagnosis tests. For example, in the differential diagnosis of coma patients, rapid automated analysis of CSF samples can benefit physicians’ quick decision making [5]. Prompt detection of malignant cells in body fluid samples including bloods may be useful for the diagnosis of disseminated intravascular coagulation [6].

Although manual microscopic examinations are most widely used in hematology laboratories, those are time consuming and results are sometimes hampered by inter-examiners’ variability in their skill levels. To date, many scientists and industries have been attempting to develop automated analyzing systems, and several different algorithms of the automated hematology analyzers have been developed to count and differentiate nucleated cells in various BF samples such as synovial, cerebrospinal, pleural, ascitic and pericardial fluids [7–10]. However, detection of malignant cells in BF samples by the hematology analyzers is still challenging because cell size, shape and cytoplasmic density of malignant cells vary as well as malignant cells often stick each other and form cell clumps.

Recently, a new detection mode, called high-fluorescence body fluid (HF-BF) [8, 11], has been equipped to the automatic hematoanalyzer Sysmex XN series (Sysmex, Kobe, Japan) perusing to discriminate non-haematopoietic cells. However, the nonmalignant cells such as mesothelial cells or macrophages are counted as the HF-BF cells along with malignant cells, and current HF-BF based analysis still causes false-positive results frequently. Thus, further improvement of the HF-BF to realize more accurate detection of malignant cells by modification of its parameter setting are warranted.

In this study, we propose a new XN-BF gating algorithm to detect malignant cells by modification of the conventional HF-BF algorithm. Specifically, two gating parameters, Rule 1 and Rule 2, based on the WDF channel were combined with HF-BF: (1) Rule 1 detects signals from large cells and clumped cells of which the most cells are consisted of clustered malignant cells; and (2) Rule 2 detects middle sized mononuclear cells with less granules than neutrophils and similar fluorescence signal to monocytes of which the most cells are consisted of hematological malignant cells and solid tumor cells. This novel algorithm was tested using various BF samples with and without malignant cells, and was evaluated by comparing with the cytological diagnosis in pathology laboratory.

## Materials and methods

### Patient samples

Between August 2013 and July 2014, a total of 92 BF samples were sent from different departments to the clinical laboratory of Juntendo University Hospital (Tokyo, Japan). The samples included 18 cerebrospinal fluid (CSF), 68 pleural effusion (PE) and 6 ascitic fluids for routine diagnosis of malignancy. The samples were stored in K_2_-EDTA tubes or sterile recipients without anticoagulant and requested to be sent to the laboratory immediately after collection. After arrival, automated analysis, manual microscopic WBC count and preparation of slides for microscopic cell differentiation were completed in hematology laboratory within two hours. Cytological examinations were performed at the pathology laboratory for these 92 BF samples. The study protocol was approved by the institutional review board of Juntendo University Hospital. Since all samples were de-identified, providing written informed consents to each patient was waved by the committee.

### Cell analyses by BF mode on the Sysmex XN hematology analyzer

The XN-series analyzers are fully automated hematology analyzers equipped with dedicated BF measurement modes. To count and differentiate nucleated cells in BF, the cell membranes are perforated with Lysercell WDF^TM^ (Sysmex, Kobe, Japan), then intracellular organelles and nucleic acids are stained with Fluorocell WDF^TM^ (Sysmex, Kobe, Japan) so that the fluorescence flow cytometric analyses can detect specific side scattered signals which are generated depending on the type and quantity of intracellular organelles and nucleic acids. After treatments with these buffers, leukocytes rarely aggregate, and platelets are usually lysed (data not shown). Very small residual platelet and fibrin aggregates are almost negligible to affect forward scatter signals.

All BF samples were first analyzed by the BF mode of the XN-1000 Sysmex hematology analyzer (XN-BF) according to the manufacture’s protocol. If clots or fibrins were observed, they were manually removed. The XN-BF mode utilizes the WBC differential (WDF) channel that simultaneously generates four signals of each cell passing through the focused laser beam in Flowcell (i.e., detecting chamber): (1) forward scatter signals indicating the volume of the cell; (2) side scatter signals providing the information about intracellular structures and contents such as nucleus and granules; (3) fluorescence intensity signals indicating the amount of intracellular nucleic acids (i.e., DNA and RNA) presenting in the cell; and (4) the forward scatter width signals providing the “time of flight” implying the doublets or highly aggregated cells passing through. The combination of these four signals of each cell depicts “scattergram” with which cells are analyzed and categorized into subgroups by using software algorithms.

Fluorescence signals were utilized to differentiate WBC from the non-haematopoietic HF-BF cells showing higher fluorescence intensity than the cutoff value. HF-BF cells were not included in the WBC counts and the amounts of HF-BF cells were expressed as a ratio over the WBCs (HF-BF/100 WBCs, abbreviated as ‘HF-BF%’) or absolute cell counts (number of HF-BF/μL, abbreviated as ‘HF-BF#’). The total nucleated cell count (TNC) is the sum of the number of WBCs and HF-BF cells. Each type of WBCs was classified as following: neutrophils and eosinophils are counted as polymorphonuclear (PMN) cells; lymphocytes and monocytes are counted as mononuclear (MN) cells; and mesothelial cells, macrophages and malignant cells are counted as HF-BF cells.

### Novel scattergram gating algorithm

**Fig 1:**
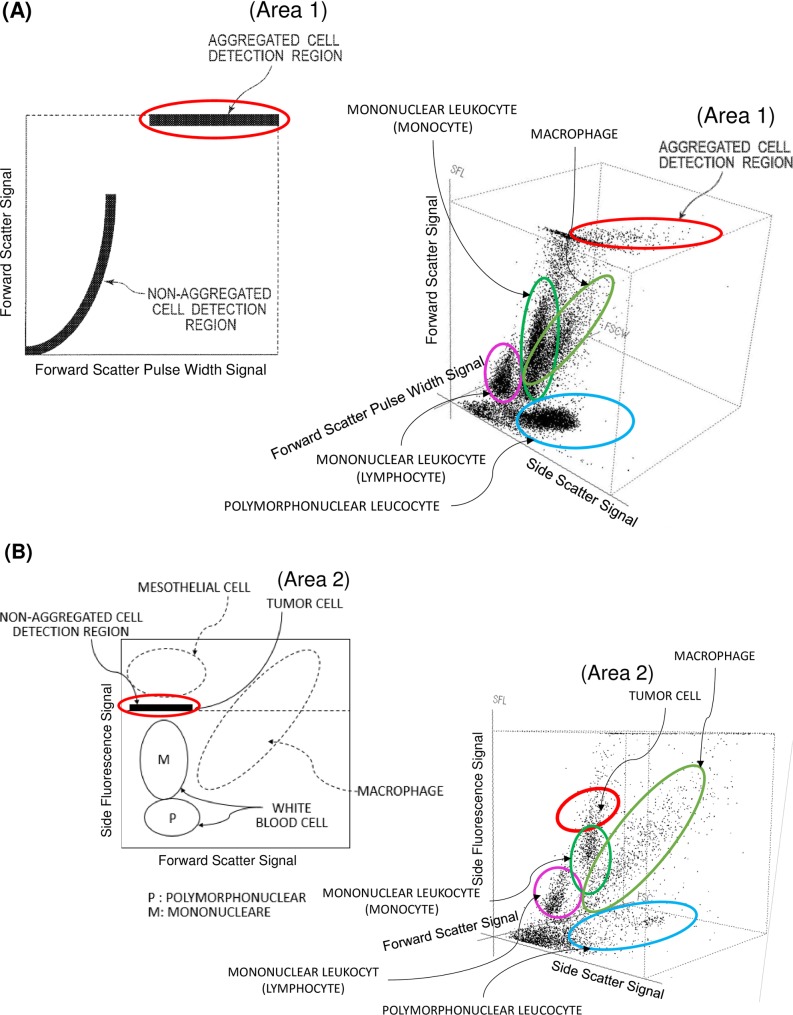
Schematic illustration of the XN-BF gating algorithm. (A) Area 1 includes the aggregated cells due to the high forward scatter width signals that implies the long “time of flight” of the cells passing though the laser beam in the flow-cell. The HF-BF cells gated in Area 1 are considered as the Rule1 positive cells. The horizontal axis indicates forward scatter pulse width signal and the vertical axis shows forward scatter signal in the 2D scattergram. In the 3D scattergram, X-axis shows side scatter signal, Y-axis is forward scatter signal and Z-axis indicates forward scatter pulse width signal. (B) Area 2 includes the non-aggregated HF-BF cell showing relatively low fluorescent signal intensity, which are defined as the Rule 2 positive cells. The horizontal axis indicates forward scatter signal and the vertical axis shows side fluorescence signal in the 2D scattergram. In the 3D scattergram, X-axis indicates side scatter signal, Y-axis shows side fluorescence signal and Z-axis is forward scatter signal. The gating illustrated based on the plotting WDF channel dot plots.

**Fig 2:**
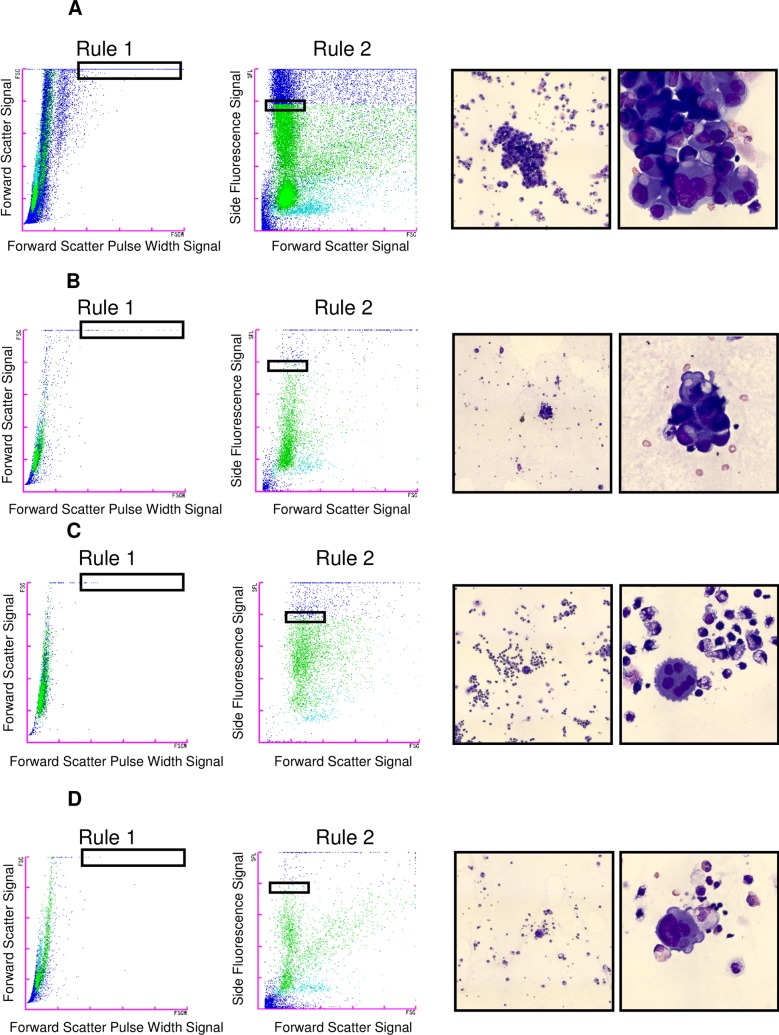
Representative analyses of body fluid samples by the XN-BF gating algorithm and photomicrographs of their corresponding cytospins. Representative XN-BF scattergrams (Rule1 and Rule2) and pictures of cytospin slides of the exact same samples (May-Grunwald-Giemsa, original magnification x10 and x50). (A) PE with adenocarcinoma cells (sample #4, Table 2). Rule 1 positive and Rule 2 positive by the XN-BF gating algorithm. (B) PE with adenocarcinoma cells (sample #13, Table 2). Rule 1 positive, but Rule 2 negative by the XN-BF gating algorithm. (C) PE with malignant mesothelioma cells (sample #16, Table 2) Rule 1 negative, but Rule 2 positive by the XN-BF gating algorithm. **(**D) PE with adenocarcinoma cells (sample #18, Table 2) interpreted as negative for malignancy by the XN-BF gating algorithm (Rule 1 negative and Rule 2 negative).

The analyzed signals of the XN-BF were exported as FCS dataset for plotting WDF channel dot plots, or scattergrams. The analyses were performed using Flowing Software (Centre for Biotechnology, University of Turku, Finland: http://flowingsoftware.btk.fi/) [12]. Two following gating algorithm based on the WDF channel were combined: (1) Rule 1 was designed to detect the aggregated cells expressing higher forward scatter width signal and higher fluorescence signals than WBCs (as indicated in Area 1, Fig 1A); (2) Rule 2 was designed to detect middle range of fluorescence signals between WBCs and HF-BF cells with lower forward scatter signal compared to macrophages. This can differentiate the isolated malignant cells with similar size but generating higher fluorescence signals compared to the normal mononuclear cells (as indicated in Area 2, Fig 1B). BF samples that meet, at least, one criterion were detected as malignant cells.

### Manual microscopic analyses

A hemocytometer (Fuchs-Rosenthal Rotterdam, Netherlands) was used for manual microscopic cell counting. For cell differentiation, cytospin slides were prepared by cytocentrifugation at 80 x g for 10 min. BF with high TNC (>1000 cells/μL) were diluted with Phosphate Buffered Saline (PBS) to obtain a concentration of 500–1000 cells/μL. Slides were stained by May-Grunwald-Giemsa. All slides were microscopically examined and performed differential count for 200 cells by an experienced medical technologist who was educated in hematopathology and cytopathology and a senior clinical pathologist. BF collection and analyses were performed in compliance with the CLSI H56-A guideline [13]. Concurrent pathological examinations were performed with Papanecolou (PAP) stained slides, followed by immunocytochemistry at an in-house pathology laboratory. Malignant cells were defined as ≥ Class IIIb (Papanicolaou class system) by the cytological examination.

### Statistics

The accuracy to detect malignant cells among various methods were evaluated by Mann-Whitney U-test where appropriate. *p*<0.05 was considered as statistically significant.

## Results

### Comparison of the accuracy of the XN-BF analysis and the microscopic examinations in the hematology laboratory compared to the examinations in the cytology laboratory

Table 1 summarizes the results of automated analyses and manual microscopic examinations. In the 92 BF samples, malignant cells were detected in 27 samples by the cytology examination. Although these two new algorithms (i.e., Rule 1 and Rule 2) are supposed to work complementally, there are some difference in the accuracy in the detection of malignant cells. Of the 27 malignant samples, five samples were detected by Rule 1 and 2 (18.5%), nine samples were detected by Rule 1 alone (33.3%), three samples were detected by Rule 2 alone (7.40%) and nine samples were not detected as malignant by either method (33.3%).

**Table 1:** Characteristics of BF samples.

Sample #	Material	Pathology		XN-BF data				Malignancy			
		Histological cancer type	Class	Total cell #	WBC#	HF-BF#	HF-BF%	XN algorism		Microscopic examination	
				(/μL)	(/μL)	(/μL)	(%)	Rule1	Rule 2	WBC differentiation	WBC count
1	PE†	Adenocarcinoma	V	1828	998	830	83.2	+	+	+	+
2	PE	Adenocarcinoma	V	1241	771	470	61.0	+	+	+	+
3	PE	Adenocarcinoma	V	2460	1623	837	51.6	+	+	-	+
4	PE	Adenocarcinoma	V	3597	2526	1071	42.4	+	+	+	+
5	PE	Adenocarcinoma	V	3424	2723	701	25.7	+	+	+	+
6	PE	Lung Adenocarcinoma	V	1469	342	1127	329.5	+	-	+	+
7	PE	Malignant Lymphoma	V	1333	612	721	117.8	+	-	+	+
8	PE	Adenocarcinoma	V	1528	1018	510	50.1	+	-	-	+
9	PE	Adenocarcinoma	V	353	271	82	30.3	+	-	+	+
10	AF‡	Adenocarcinoma	V	124	100	24	24.0	+	-	+	+
11	PE	Small cell carcinoma	V	2124	1733	391	22.6	+	-	+	+
12	AF	Adenocarcinoma	V	1404	1160	244	21.0	+	-	+	+
13	PE	Adenocarcinoma	V	385	344	41	11.9	+	-	+	+
14	PE	Lung Adenocarcinoma	V	1391	1320	71	5.4	+	-	+	+
15	PE	Adenocarcinoma	V	218	124	94	75.8	-	+	+	+
16	PE	Malignant Mesothelioma	V	426	358	68	19.0	-	+	+	+
17	PE	Adenocarcinoma	V	609	494	115	23.3	-	-	-	-
18	PE	Adenocarcinoma	V	297	277	20	7.2	-	-	+	-
19	PE	Adenocarcinoma	IIIb	1682	1590	92	5.8	-	-	-	-
20	PE	Adenocarcinoma	V	2773	2647	126	4.8	-	-	+	+
21	PE	Adenocarcinoma	V	386	376	10	2.7	-	-	-	-
22	PE	Lung Adenocarcinoma	V	3888	3806	82	2.2	-	-	+	-
23	PE	Malignant Lymphoma	V	2927	2885	42	1.5	-	-	-	-
24	PE	Adenocarcinoma	V	3786	3737	49	1.3	-	-	-	-
25	AF	Adenocarcinoma	V	207	182	25	13.7	-	-	-	-
26	CSF§	unknown	V	909	373	536	143.7	-	+	+	+
27	CSF	Medullobrastoma	V	53	50	3	6.0	-	-	+	-
28	PE	N/D¶	Ⅱ	5692	4977	715	14.4	+	+	+	+
29	PE	N/D	Ⅱ	5458	5240	218	4.2	+	-	-	-
30	PE	N/D	Ⅱ	2785	2558	227	8.9	+	-	-	-
31	PE	N/D	Ⅱ	1203	985	218	22.1	-	+	+	+
32	PE	N/D	Ⅱb	2106	1825	281	15.4	-	+	-	-
33	PE	N/D	Ⅱ	106	84	22	26.2	-	+	-	-
34	PE	N/D	Ⅱ	217	193	24	12.4	-	+	-	-
35	PE	N/D	Ⅱ	261	219	42	19.2	-	+	-	-
36	PE	N/D	Ⅲ	89	85	4	4.7	-	-	-	+
37	PE	N/D	Ⅱ	949	856	93	10.9	-	-	-	+
38	PE	N/D	Ⅱ	3596	3500	96	2.7	-	-	-	+
39	PE	N/D	Ⅲ	949	816	133	16.3	-	-	-	-
40	PE	N/D	Ⅲ	48	45	3	6.7	-	-	-	-
41	PE	N/D	Ⅲ	1008	994	14	1.4	-	-	-	-
42	PE	N/D	Ⅲ	307	287	20	7.0	-	-	-	-
43	PE	N/D	Ⅲ	242	219	23	10.5	-	-	-	-
44	PE	N/D	Ⅲ	6760	6647	113	1.7	-	-	-	-
45	PE	N/D	Ⅲ	7037	6668	369	5.5	-	-	-	-
46	PE	N/D	Ⅱb	208	174	34	19.5	-	-	-	-
47	PE	N/D	Ⅱb	1114	1031	83	8.1	-	-	-	-
48	PE	N/D	Ⅱ	59	58	1	1.7	-	-	-	-
49	PE	N/D	Ⅱ	36	34	2	5.9	-	-	-	-
50	PE	N/D	Ⅱ	359	357	2	0.6	-	-	-	-
51	PE	N/D	Ⅱ	609	606	3	0.5	-	-	-	-
52	PE	N/D	Ⅱ	706	702	4	0.6	-	-	-	-
53	PE	N/D	Ⅱ	1914	1907	7	0.4	-	-	-	-
54	PE	N/D	Ⅱ	5216	5207	9	0.2	-	-	-	-
55	PE	N/D	Ⅱ	193	173	20	11.6	-	-	-	-
56	PE	N/D	Ⅱ	239	217	22	10.1	-	-	-	-
57	PE	N/D	Ⅱ	842	817	25	3.1	-	-	-	-
58	PE	N/D	Ⅱ	1753	1728	25	1.4	-	-	-	-
59	PE	N/D	Ⅱ	208	178	30	16.9	-	-	-	-
60	PE	N/D	Ⅱ	273	243	30	12.3	-	-	-	-
61	PE	N/D	Ⅱ	6651	6620	31	0.5	-	-	-	-
62	PE	N/D	Ⅱ	1014	972	42	4.3	-	-	-	-
63	PE	N/D	Ⅱ	6632	6578	54	0.8	-	-	-	-
64	PE	N/D	Ⅱ	509	452	57	12.6	-	-	-	-
65	AF	N/D	Ⅱ	1271	1197	74	6.2	-	-	-	-
66	PE	N/D	Ⅱ	777	699	78	11.2	-	-	-	-
67	PE	N/D	Ⅱ	1945	1838	107	5.8	-	-	-	-
68	PE	N/D	Ⅱ	2354	2234	120	5.4	-	-	-	-
69	PE	N/D	Ⅱ	2232	2107	125	5.9	-	-	-	-
70	AF	N/D	Ⅱ	548	399	149	37.3	-	-	-	-
71	PE	N/D	Ⅱ	1736	1470	266	18.1	-	-	-	-
72	PE	N/D	Ⅱ	3891	3539	352	9.9	-	-	-	-
73	PE	N/D	Ⅱ	3148	2746	402	14.6	-	-	-	-
74	AF	N/D	Ⅱ	268	253	15	5.9	-	-	-	-
75	PE	N/D	Ⅱ	1166	1143	23	2.0	-	-	-	-
76	PE	N/D	Ⅱ	1127	1096	31	2.8	-	-	-	-
77	CSF	N/D	Ⅲ	2	2	0	0.0	-	-	-	-
78	CSF	N/D	Ⅲ	8	8	0	0.0	-	-	-	-
79	CSF	N/D	Ⅲ	112	106	6	5.7	-	-	-	-
80	CSF	N/D	Negative	1	1	0	0.0	-	-	-	-
81	CSF	N/D	Negative	1	1	0	0.0	-	-	-	-
82	CSF	N/D	Negative	1	1	0	0.0	-	-	-	-
83	CSF	N/D	Negative	2	2	0	0.0	-	-	-	-
84	CSF	N/D	Negative	2	2	0	0.0	-	-	-	-
85	CSF	N/D	Negative	2	2	0	0.0	-	-	-	-
86	CSF	N/D	Negative	11	10	1	10.0	-	-	-	-
87	CSF	N/D	Negative	7	7	0	0.0	-	-	-	-
88	CSF	N/D	Negative	246	241	5	2.1	-	-	-	-
89	CSF	N/D	Negative	115	115	0	0.0	-	-	-	-
90	CSF	N/D	Negative	23	22	1	4.5	-	-	-	-
91	CSF	N/D	Negative	5	5	0	0.0	-	-	-	-
92	CSF	N/D	Negative	10	9	1	11.1	-	-	-	-

†PE, pleural effusion

‡AF, ascitic fluid

§CSF, cerebrospinal fluid

¶N/D, not detected

The accuracy of the XN-BF gating algorithm as well as the manual microscopic examinations in hematology laboratory in the detection of malignant cells were evaluated by a comparison with the cytological diagnosis. Table 2 summarized the results of accuracy by the three types of examinations performed in hematology laboratory showed high specificities (87.7–96.9%) but relatively low sensitivities (63.0–70.4%), with the highest accuracy of the WBC differential examination with May-Grunwald-Giemsa staining. We, then, analyzed the difference between true positive and false negative with the XN-BF gating algorithm in the cytology positive malignant BF samples. As shown in Table 3, the false negative samples contained significantly lower number of HF-BF cells (*p* = 0.008) than the true positive samples, which indicates that a small malignant cells number is at least one of the causes of the false negative with the XN-BF gating algorithm. No significant difference was observed among these methods in total cell numbers or WBC numbers.

**Table 2:** Performance of examinations in hematology laboratory in the detection of BF malignancy compared with cytological diagnosis.

Type of examination	Sensitivity (%)	Specificity (%)	Positive Predictive Value (%)	Negative Predictive Value (%)
XN-BF gating algorism	63.0	87.7	68.0	85.1
Microscopic WBC differential (May-Grunwald Giemsa stain)	70.4	96.9	90.5	88.7
Microscopic WBC count (Fuchs-Rosental)	66.7	92.3	78.3	87.0

**Table 3:** Sysmex XN-BF data and performance of examinations in hematology laboratory to detect malignancy.

XN-BF data	XN-BF gating algorism			Microscopic WBC differential		Microscopic WBC count		
	True positive (n = 15)	False negative (n = 11)		True positive (n = 18)	False negative (n = 8)	True positive (n = 17)	False negative (n = 9)	
Total cell # (/μL)	1457 ± 265	1592 ± 448		1485 ± 281	1698 ± 455	1499 ± 248	1537 ± 530	
WBC # (/μL)	1001 ± 203	1492 ± 452		1108 ± 251	1488 ± 447	1058 ± 206	1489 ± 525	
HF-BF # (/μL)	455 ± 96	100 ± 45	*p* = 0.008	377 ± 84	210 ± 106	441 ± 87	49 ± 13	*p* = 0.004
HF-BF % (%)	61±19	19 ± 13		54 ± 17	19 ± 7	62 ± 18	7 ± 2	*p* = 0.044

The data are presented as mean ± SEM.

### The examples in the detection of malignant cells by the new algorithms

As described in the methods, the XN-BF gating algorithm is supposed to detect malignant cells with relatively large size and/or cell clumps by Rule 1, and cells contains condensed intracellular contents such as RNAs and DNAs are supposed to be detected by Rule 2. Fig 2 shows representative scattergrams of XN-BF and corresponding May-Grunwald-Giemsa staining photomicrographs. Fig 2A through C show representative body fluids with true positive results of the XN-BF gating algorithm. Malignant cells detected by Rule 1 and/or Rule 2. Fig 2A shows a malignant PE (sample #4, Table 1) detected as malignancy by the XN-BF gating algorithm (positive by both Rule 1 and 2) containing highly aggregated adenocarcinoma cells (88.7% by microscopic differential count). Fig 2B shows a malignant PE (sample #13, Table 1) detected as malignancy (Rule 1 positive, but Rule 2 negative) with the isolated cluster of adenocarcinoma cells (11.8% by the microscopic differential count). Fig 2C is a malignant mesothelioma PE (sample #16, Table 1) detected as malignancy (Rule 1 negative, but Rule 2 positive) containing malignant cells with isolated and sparse form (7.6% by the microscopic differential count). Fig 2D shows a malignant PE (sample #18, Table 1) interpreted as negative for malignancy by the XN-BF gating algorithm (negative by both Rule 1 and 2) containing small number of isolated adenocarcinoma cells (1.5% by the microscopic differential count).

## Discussion

In this study, we investigated the accuracy of a newly developed XN-BF gating algorithm in the detection of malignant cells using Sysmex XN-1000 automated hematology analyzer. As described in the method section, this algorithm was designed to detect malignant cells by addition of two detection rules to the conventional Sysmex XN-BF mode: (1) measuring passing time that is affected by cell clumps; and (2) measuring fluorescence intensity reflected by cytoplasmic organelles and nucleus contents. Whereas the sophisticated algorithms are developed for automatic computational algorithms of flowcytometry [14], our gating analyses were performed manually and our system is not using various CD markers or other special staining. This study is attempting to develop a relatively simple and more accurate detection system compared to the conventional manual microscopic examination that performed by simple May-Grunwald-Giemsa staining in hematology laboratories rather than full cytology examination in pathology laboratories at stage.

We and others have reported the difficulties to distinguish malignant cells from benign ones containing macrophages and mesothelial cells by the conventional Sysmex HF-BF mode because of its low specificity [8, 13]. This is mainly due to the use of only two detection algorithms: (1) forward scattering signal detection that can evaluate size of cells; and (2) side scattering signal detection algorithm that can detect the reflection of laser beams by cyto organells. Recently, Labaere et al. analyzed BF samples by HF-BF mode of Sysmex XN-2000 with a cut off level of ≥17 HF-BF cells /μL to detect malignant cells, and reported still relatively low specificity of 61% compared to the conventional microscopic examination with May-Grunwarld Giemsa stained slides performed in hematology laboratory [8]. Malignant cells show several unique features compared to normal blood cells: (1) these cells are usually larger than normal hematocytes; (2) these cells contain more DNAs and RNAs than normal hematocytes [15]; (3) these cells’ cytoorganelles are often more complicated than non-malignant cells; and (4) these cells have tendency to form cell clumps. We, therefore, developed a new algorithm by addition of two parameters, Rule 1 and Rule 2, to detect malignant cells more specifically, and achieved markedly high specificity of 87.8% compared to the conventional microscopic differential count with May-Grunwarld Giemsa stain in hematology laboratory. This means that the XN-BF gating algorithm reduces unnecessary microscopic analyses. On the other hands, we observed false negative results in the cases with small number of malignant cells. Further studies using more samples obtained from various kinds of body fluid samples are warranted to validate our current estimation. We are planning to expand our study to incorporate data from multiple centers, and adding more developed technology such as automated digital morphological analyzer, DI-60 which is equipped with Sysmex XN series. The combinational usage of automated high definition microscopy images may improve both specificity and sensitivity. The number of similar studies using XN-series automated analyzers is rapidly increasing all over the world. Since 2012, more than 100 studies using the XN-series have been published (referred in PubMed), and the number of studies is increasing year-by-year. To date, more than 20,000 XN-series hematology analyzers were shipped to all over the world including approximately 5000 of XN analyzers to the U.S. Thus, we believe that the XN-series analyzers have been used in many laboratories as automated hematology analyzer.

In this study, we observed that the tested different hematological examinations, including the XN-BF gating algorithm and the manual microscopic examinations in hematology laboratory, showed similar sensitivity and specificity compared to the cytological examinations. Whereas the accuracy of the XN-BF gating mode needs to be improved to attain higher sensitivity, this system might facilitate a feature upgrade of the automated hematology analyzer to detect malignant cells in BF.

As the study limitations, this is a single center study with relatively small size samples. The number of samples and type of malignant cells for each type of BF were limited. Although we examined cells from same samples for automated analyses and microscopic examinations in hematology laboratory, it was not possible to analyze exact the same cells for cytopathology laboratory since it is not feasible to collect a single cell from flow cytometry to fix and stain for cytopathological examinations. In addition, microscopic examinations by interexaminers’ and/or intraexaminers’ inconsistency could not be completely removed even though two well-trained examiners performed the studies. These issues cannot be avoided in any laboratories, and we are increasing number of samples for each kind of BF for the future study.

In conclusion, the XN-BF gating algorithm for the BF malignancy diagnosis may have a potential to be the alternative method to the morphological examination, which can benefit for hematology laboratories to screen malignant cells rapidly without requiring additional sample preparation procedure and with minimal operator bias. Currently, manual microscopic examination is the golden standard. However, in the future, we believe that accuracy and quality of automated analyses of BF samples can be further improved with the technology development, and that this type of automated detecting system can serve as an adjunct quality control system.

## Supporting information

S1 File Corresponds to the raw data for scattergrams in Fig 2A. (FCS)

S2 File Corresponds to the raw data for scattergrams in Fig 2B. (FCS)

S3 File Corresponds to the raw data for scattergrams in Fig 2C. (FCS)

S4 File Corresponds to the raw data for scattergrams in Fig 2D. (FCS)
